# Health systems appraisal of the response to antimicrobial resistance in low‐ and middle‐income countries in relation to COVID‐19: Application of the WHO building blocks

**DOI:** 10.1111/1758-5899.13262

**Published:** 2023-08-22

**Authors:** Jay Patel, Genevie Fernandes, Ambele Judith Mwamelo, Devi Sridhar

**Affiliations:** ^1^ Global Health Governance Programme Usher Institute, University of Edinburgh Edinburgh UK; ^2^ Faculty of Medicine and Health, University of Leeds Leeds UK

## Abstract

COVID‐19 has inflicted both beneficial and damaging effects on health systems responding to antimicrobial resistance (AMR). Data shows that the positive impacts of the pandemic (including enhanced hygiene, mask wearing and widespread use of personal protective equipment), are likely to have been overshadowed by the negative effects: emerging AMR pathogens and mechanisms; further outbreaks and geographic spread of AMR to non‐endemic countries; rising infections from multidrug‐resistant pathogen; an overall higher burden of AMR. The multisectoral complexities of AMR and the totality of health systems challenge our ability to understand the impact of the COVID‐19 pandemic on country responses to AMR. In this analysis, we synthesise international evidence characterising the role of the pandemic on the six key building blocks of health systems in responding to AMR across low‐ and middle‐income countries (LMICs). We apply systems thinking within and between the building blocks to contextualise the impact of one pandemic on another.

## 1 OVERLAPPING PANDEMICS

Antimicrobial resistance (AMR) is projected to cause 10 million deaths a year by 2050 and has been a major global health emergency even before COVID‐19 (Pan American Health Organization, 2021). The notion that ‘no one is safe until everyone is safe’ has defined advocacy for vaccine equity in the COVID‐19 pandemic, and equity features as a stand‐alone item in the WHO pandemic treaty working draft. Equity and the increasing recognition of our cross‐sectoral interconnectedness should similarly define strengthened global coordination for AMR governance. AMR exposes stark inequities between high‐income countries (HICs) and LMICs: lack of access to antimicrobials juxtaposes excess use of antimicrobials; suboptimal hand hygiene contrasts a lack of opportunity for hand hygiene; rapid, granular, high‐quality surveillance systems in low‐burden regions is contrary to lower quality surveillance systems in high‐burden regions.

Historically, international attention on AMR has cyclically waxed and waned, in response to the relative urgency of this public health crisis against other policy priorities (Overton et al., 2021). COVID‐19 has ushered renewed public and political interest in infectious diseases and global health security. The pandemic also enhanced awareness and practices of infection prevention and control measures. Collectively, these ripen the conditions to galvanise action on AMR both through high‐level coordination and through community engagement.

According to the Tripartite AMR Country Self‐Assessment Survey 2020–21, only 11 of 163 countries (Djibouti, Egypt, Somalia, Armenia, Bulgaria, the Netherlands, Russia, San Marino, Turkmenistan and Mongolia) did not ascribe the COVID‐19 pandemic as having significantly impacted the national response to tackling AMR, and the development or implementation of their AMR national action plan (NAP) (FAO, WOAH & WHO, 2021). In this analysis, we synthesise international evidence characterising the role of the pandemic on the six key building blocks of health systems in responding to AMR across LMICs. As the burden of drug‐resistant infections is increasingly exacerbated by the COVID‐19 pandemic, there is an urgent need to understand the impact on health systems in LMICs and prioritise efforts for its containment.

## 2 MATERIALS AND METHODS

Firstly, a comprehensive literature search was conducted for peer‐reviewed scoping, narrative, integrative or systematic reviews, exploring the impact of Covid‐19 on antimicrobial resistance in LMICs, on Google Scholar and PubMed from the database inception to April 1, 2022. We were unable to locate any reviews, validating the novelty of a scoping review investigating this area. A scoping literature review of Google Scholar and PubMed databases from January 2020 to May 2022 using keyword searches identified relevant literature. We used the search terms “antimicrobial resistance” or “antibiotic resistance” or “drug resistance” or “drug‐resistant infection*” or “AMR”, and “health system*”, and “COVID*” or “coronavirus”, “pandemic”. Although the search strategy was not restricted, we prioritised articles published in the English language; those relating to the economic context of LMICs, and those that made specific reference to the COVID‐19 pandemic. Any article types were screened, including data found online. We chose not to register the scoping review, to enable greater flexibility in the breadth of insights that could be drawn and to avoid a restrictive search framework, given the scarcity of published evidence pertaining to this area. We applied the WHO Building Blocks as a framework, to identify the functions of a health system. The search was scoping in nature, rather than systematic, to identify and collate a broader range of evidence and synthesise evidence pertaining to the six building blocks. The available evidence was mapped against the health systems components by two researchers (JP and GF). Using the collated evidence, this research article considers each of the six WHO building blocks and discusses each function in the context of LMICs.

## 3 RESULTS

The results demonstrated a scarcity of peer‐reviewed articles pertaining to the role of the COVID‐19 pandemic on health systems responding to AMR in LMICs. The evidence evaluated largely originated from the grey literature and online sources that were corroborated among the research team to identify the key findings.

### 3.1 Health service delivery

Overseas developmental assistance for health (inputs) and service delivery (outputs) demonstrate the complex relationship between global agencies and local service delivery. Conventionally, global health funding has been channelled towards vertical donor interventions, which often do not contribute towards strengthening national health systems holistically, but aggressively focus on targeted solutions for specific disease priorities. While this approach can be highly effective for the management of single infectious diseases, the enhancement of an overall disease control capacity can yield greater benefits. Only 2% of global health funding is spent on health systems strengthening (Alliance for Health Policy and Systems Research, 2021). Without an emphasis on health systems strengthening, the basis for achieving the service delivery characteristics for AMR is lacking, requiring more complex and expensive interventions at the global level. Due to the multisectoral nature of AMR and One Health, a vertical programmatic approach may not present adequate utility. During the pandemic, shortcomings in country responses were attributable to inadequate health service delivery at the national and global levels. Repeating the mistakes of limited resource inputs and underinvesting in strengthening national health systems risk worsening the burden of AMR, and the ability to formulate a response.

Health service disruptions, diagnostic delays, longer hospital inpatient stays from COVID‐19, weak infection control and prevention measures and delayed detection of AMR outbreaks may have contributed to increased AMR transmissions during the pandemic (Knight et al., 2021). A review of data from COVID‐19 cases, mostly in Asia, found that more than 70% of patients received antimicrobial treatment despite less than 10%, on average, having bacterial or fungal coinfections (Rawson et al., 2020). In high AMR burden settings, excessive use of hygienic products, practice of self‐medication and expanded production of antimicrobials associated with COVID‐19, could amplify the concentration of these compounds in the environment (Seethalakshmi et al., 2022). This is further challenged by weak waste management infrastructure and poor sanitation observed in certain LMICs. Among hospitalised COVID‐19 patients in India, a predominance of Gram‐negative bacteria resistant to a higher generation of antimicrobials was observed (Vijay et al., 2021).

The challenges relating to health coverage in LMICs can be broadly summarised as functional and infrastructural challenges. Without optimal health coverage, particularly in communities in rural areas, timely access to (initially narrow‐spectrum) antibiotics is compromised. Equally, the absence of restrictive policies to prevent inappropriate prescribing can result in situations where excessive use is commonplace. However, we recognise that restrictive regulatory and legislative policy alone may not be sufficient to prevent inappropriate prescribing, as even in nations where such policies exist, the misuse of antimicrobials remains prevalent (FAO, WOAH & WHO, 2021). Given that inappropriate prescribing is a widely recognised driver of AMR, this highlights the urgent need for robust enforcement of policies, ensuring adequate capacity to effectively curb inappropriate prescribing practices. Universal health coverage in low‐resource contexts should not be equated to the availability of antimicrobial agents, but the holistic health service delivery, including the coverage of health care workers, who can responsibly prescribe these medicines.

Constrained economic growth, poor fiscal management and low preparedness in some LMICs distract the necessary focus on basic developmental matters. Enhancing infection prevention and control measures, including clean water, sanitation and hygiene initiatives, is central to sustainable development. Yet for political purposes, antibiotics are quick, easily deployable and cheap solutions to conceal these deficiencies in infrastructure planning and investment decisions. This has been a concerning side effect of mass drug administration campaigns for antibiotics and anthelmintics to treat childhood pneumonia in Pakistan (Bogoch et al., 2019). Universal and equitable access to safe drinking water and sanitation could yield a 60% reduction in diarrhoeal diseases treated with antimicrobials—averting the need for 300 million courses of antibiotics in four middle‐income countries alone (India, Indonesia, Nigeria and Brazil) (O'Neill, 2016). Encouraging and financing these basic and foundational improvements in the poorest countries will pay dividends far beyond AMR, with major social and economic benefits.

### 3.2 Health workforce

The COVID‐19 response demonstrated the value of evidence‐based behavioural interventions for population‐wide adherence to public health measures. Where locally‐delivered and context‐specific interventions were supportively applied, public compliance improved due to the perceived increase in interpersonal and institutional trust. Similarly, this behavioural understanding can be applied to AMR to inform evidence‐based solutions.

Areas where COVID‐19 and AMR overlap and align should be identified and re‐inforced, for example, training and education, broader interdisciplinary teams and communities of practice (Tomczyk et al., 2021). Reductions in the availability of nursing, medical and public health staff for AMR were reported by 71%, 69% and 64%, respectively, leading to delayed detection of AMR cases and clusters (Tomczyk et al., 2021). Interventions to improve antibiotic prescribing are grounded in the capacity for society‐wide behaviour change. Pharmacy personnel are an under‐recognised and underused dimension of the health workforce in driving antimicrobial stewardship across LMICs. Transposing their value over the COM‐B model for behaviour change elicits their potential value (Michie et al., 2011). Pharmacists are uniquely positioned to contribute towards stewardship and better infection prevention and control practices through three domains: (1) capability (specialist knowledge of medicines, relevant decision‐making abilities, interpersonal skills); (2) opportunity (contact with prescribers and patients, ability to deliver guidance, share knowledge and promote vaccination uptake); (3) motivation (inherent professional commitment to the rational use of medicines) (Ashiru‐Oredope et al., 2022).

### 3.3 Health information systems

Surveillance is essential for harnessing health information on AMR. Major advances have been made in the international surveillance of AMR, including estimates of the global burden of disease attributable to and associated with bacterial drug‐resistant infections (Antimicrobial Resistance Collaborators, 2022); the prevalence and incidence of resistant organisms (World Health Organization, 2022); antimicrobial use data in animals (World Organization for Animal Health, 2022). At present, these datasets vary in their comprehensiveness; systematicity, in terms of measurement methods; contemporaneity. In particular, the estimation of the true burden of disease through accurate surveillance methods and systems remains an area of continued development in LMICs. Constraints in health systems resources and capacities, and gaps in data are long‐standing obstacles to AMR implementation. Regrettably, these challenges have retained a centrality as defining limitations in addressing AMR, and are most pronounced in LMICs, where a lack of budgetary support limits the prioritisation of AMR‐related surveillance (Aruhomukama, 2022).

While mathematical modelling attempts to correct for data gaps—that are more pronounced in LMICs—the importance of high‐quality primary data remains critical for iterative improvements of regional situational analyses, clinical guidelines, policies and monitoring and evaluation efforts. GLASS provides an accessible and collaborative mechanism capable of addressing these data challenges, but requires financial and technical support for expansion in LMICs (World Health Organization, 2022). A defining principle of the Global Burden of Disease Study is to *leave no gaps* when generating health metrics; this reflects an interpretative propensity for policymakers to imply that data gaps relate to no disease burden, which can have serious and damaging implications for priority‐setting.

Greater consideration should also be paid to how surveillance data can be transformed to be more analytical and predictive to proactively inform policy, rather than for international informational and monitoring purposes (Kandel et al., 2022). COVID‐19 dashboards, capturing near real‐time data, have provided governments the opportunity for agile decision‐making at national and subnational levels.

In the same way that SARS‐CoV‐2 variants affected country responses, shows that surveillance needs to be wide and not just focused on a handful of priority pathogens, as is the case in most HICs.

Often, metrics used to ascertain the relative risk of an acute global public health emergency, are based upon the degree of protection conferred on high‐income and upper‐middle countries from health threats emerging in low‐income countries (Brown et al., 2022). This assumption was dismantled by following the evolutionary trajectory of SARS‐CoV‐2, where variants were first detected in high‐income settings, for example, Alpha (United Kingdom), Beta (South Africa) and Gamma (Brazil).

### 3.4 Access to essential medicines

COVID‐19 led to a surge in demand for antimicrobials (Patel & Sridhar, 2022), such as those for the treatment of rare fungal infections, which have become more common as a consequence of the pandemic. LMICs have faced difficulties in accessing antimicrobials due to shortages or regulatory implications for the incorporation of new drugs. Excessive and inappropriate use of antibiotics is compounded by the relative ease of procuring medicines without a prescription or any involvement with trained healthcare professionals. This trend was not universal, as antimicrobial sales and consumption appeared to have declined in some nations, particularly during the initial, acute phase of the pandemic (Lord, 2021).

The availability of antimicrobials has been disrupted by supply chain challenges and changes in global manufacturing, leading to alterations in usage patterns. The limited monitoring capacity for national regulatory authorities in LMICs to produce therapeutics fosters the conditions for a manufacturing environment of low‐quality and counterfeit drugs, with few or no active ingredients. As a corollary, the clinical indication for second‐ and third‐generation antibiotics is often necessitated. COVID‐19 amplified the international debate on pharmaceutical production capacities across LMICs, and the pandemic treaty is an important opportunity to achieve these goals.

Transitioning reliance on diagnosis from empirical diagnostics to rapid tests would facilitate more accurate prescribing. Of 40 million people in the United States, who are prescribed antibiotics for respirations conditions, 27 million are prescribed antibiotics unnecessarily. Diagnostics present a questionable economic case (O'Neill, 2016). Although their high‐level population‐wide benefits show clear cost‐effectiveness, at the local level, additional expenditures for diagnostic tools may not necessarily present a convincing economic case relative to empirical diagnostic methods.

A critical lesson from the pandemic is that when governments create supportive environments, science delivers (Nandi et al., 2023). The compression of vaccine development timescales from years to months was enabled by markets, technology and public‐private partnerships working collaboratively to provide solutions. Governments setting the right regulatory frameworks to encourage the necessary innovation (and avoiding overly prescriptive solutions) enabled this success.

A major supply‐side problem with AMR is the dearth of antibiotic development in recent decades and the scarcity of promising therapeutics in the pipeline. Building on the recent successes in vaccination, governments should aim to connect research and science capability with economic capacity. The partnership between Oxford University and AstraZeneca in developing the ChAdOx1 nCoV‐19 vaccine demonstrates the value of linking academic and industrial capability, and allowing governments to play a brokering role. The UK and US governments have recently endorsed initiatives leveraging pull incentives and creating market incentives to encourage novel antibiotic development (National Institute for Health Care Excellence, 2023; US Congress, 2021). These strategies decouple the financial reward from prescriptions, by contractually agreeing fixed annual fees to developers, encouraging pharmaceutical companies to develop novel antibiotics intended for last‐resort use.

### 3.5 Health systems financing

A further economic repercussion of the rise in AMR is the increased cost of new antimicrobial treatments (Pan American Health Organization, 2021). Several key challenges pertaining to the inappropriate use of antibiotics in LMICs could be more optimally managed by integrating national AMR response strategies with universal health coverage (UHC). This synergy pays dividends for LMICs in at least four major ways: (1) enhanced infection prevention and control; (2) improved access to appropriate and affordable treatments and greater awareness of their responsible use; (3) financial incentives, including the decoupling of drug prescriptions and HCW income; (4) strengthened institutional arrangements and partnerships for better regulation and stewardship (Bloom et al., 2017). When AMR is embedded in a UHC framework, the challenge of access to essential antimicrobials can be reduced. Financial schemes compensating outpatient treatments for infections would break down the existing financial barriers for patients procuring timely and appropriate medicines and preventing the progression of disease, which may necessitate broader spectrum antibiotics.

Critically, the global health system remains underfunded, preventing collective action to respond to transnational challenges. Compulsory financing to realise predictable funds to support global governance functions would support AMR control initiatives and broader public health activities. The case for investment in AMR is clear: spending $40 billion over a 10‐year period, could mitigate a cumulative global economic loss of $100 trillion by 2050, which has a similar economic framing for pandemic preparedness (Patel & Sridhar, 2021).

### 3.6 Leadership and governance

Even before COVID‐19 hit, several countries had not allocated financial budgets for implementing their national action plans to control and reduce AMR (Patel et al., 2023). With the COVID‐19 pandemic, existing political priority and resources shifted from other pressing health issues including AMR. AMR surveillance and stewardship programmes have been deprioritised in LMICs (Rodríguez‐Baño et al., 2021). Further, regulation of antibiotics has been challenging, particularly in livestock consumption (Devraj, 2019). Endorsement of certain drugs despite the lack of comprehensive data on effectiveness for treating COVID‐19 resulted in overprescription, including for prophylaxis, overpricing and shortage (Pulla, 2020). COVID‐19 may have exacerbated this challenge of inadequate regulatory systems stemming from weak governance and leadership.

The pandemic has offered an opportunity to invest in infection control. Integrating and aligning AMR in national pandemic preparedness plans is one way to refocus attention on AMR and prevent relapse on any progress made in LMICs so far. The pandemic has revealed that risk communication that is timely, consistent, transparent and from a trusted source is crucial to public engagement and acceptance. Governments will need to apply such lessons to increase the awareness of AMR risk. Innovative interventions can strengthen antimicrobial stewardship activities in community, primary and tertiary health facilities to ensure appropriate use of antimicrobials. An NIHR‐funded global health research programme tested two interventions in Bangladesh to accurately detect childhood pneumonia; first, community health worker delivered digital auscultation, and second, an artificial intelligence model to interpret chest radiographs (Ahmed et al., 2022; Sun et al., 2021). Both the interventions have demonstrated positive research findings, are feasible to implement, and when further tested and scaled up, could facilitate accurate diagnosis of childhood pneumonia and reduce misdiagnosis and the unnecessary use of antibiotics. Improved government coordination between federal, provincial and district levels as well as between public and private health system actors is urgently needed to strengthen AMR surveillance, for early detection of AMR and novel resistant mechanisms and reinforcing infection prevention and control practices.

Multisectoral stakeholder engagement, including government and non‐government actors, will be critical in mobilising resources and response for AMR programmes as well as strengthening sanitation and waste management infrastructure and influencing the practice of handwashing, disinfection and social distancing when ill. Harnessing the public understanding of the relevance of infectious diseases towards the long‐term pandemic of AMR could have major implications for promoting good practices about AMR and the control of transmission.

Community engagement and involvement will be a critical strategy for AMR researchers and practitioners. Reducing and controlling AMR in LMICS will require champions in the policy landscape, whether in federal government programmes or development partners, as well as in the community in the form of pharmacists, community health workers and leaders.

## 4 CONCLUSION

Our analysis synthesises international evidence demonstrating the effect of the COVID‐19 pandemic on each of the six components relevant to health systems in LMICs responding to AMR. Before data characterising the pandemic's role on AMR were available, speculations of both a beneficial and damaging role were widely reported. However, it is now clear that COVID‐19 has had an overall negative role on AMR, leading to a crude undoing on progress to control AMR. Governments should consider interventions to reverse this acute exacerbation as part of a post‐pandemic recovery package. If these trends are not reversed, the health, economic and social impacts of AMR may be far worse than COVID‐19 and compounding health crises for LMICs could significantly stunt their sustainable development.

## FUNDING INFORMATION

This research was funded by the Wellcome Trust (106635/Z/14/Z).

## CONFLICT OF INTEREST STATEMENT

The authors declare no competing interests.

## Data Availability

There is no data in this work.
